# Lead concentrations in commercial dogfood containing pheasant in the UK

**DOI:** 10.1007/s13280-023-01856-x

**Published:** 2023-05-03

**Authors:** Deborah J. Pain, Rhys E. Green, Nicola Bates, Maider Guiu, Mark A. Taggart

**Affiliations:** 1 Department of Zoology, University of Cambridge, Downing Street, Cambridge, CB2 3EJ UK; 2 School of Biological Sciences, University of East Anglia, Norwich Research Park, NR4 7TJ UK; 3 Centre for Conservation Science, RSPB, The Lodge, Sandy, SG19 2DL Bedfordshire UK; 4 Veterinary Poisons Information Service, 2nd Floor, Godfree Court, 29–35 Long Lane, London, SE1 4PL UK; 5 Environmental Research Institute, University of the Highlands and Islands, Castle Street, Thurso, KW14 7AP UK

**Keywords:** Dogfood, Lead, Pheasant, Poisoning, Shot, UK

## Abstract

UK and EU regulators are evaluating the potential health benefits of restricting the use of lead ammunition. Little information is available on exposure of pets to ammunition-derived dietary lead from petfood containing meat from wild-shot game animals. We found dogfood including wild-shot pheasant meat to be widely available in the UK. 77% of samples from three raw pheasant dogfood products exceeded the EU maximum residue level (MRL) for lead in animal feed, with mean concentrations approximately 245, 135 and 49 times above the MRL. Concentrations > MRL were also found in a dried food containing pheasant, but not in a processed food, nor in chicken-based products. Lead concentrations in raw pheasant dogfood considerably exceeded those in pheasant meat sold for human consumption, possibly because the dogfood mincing process further fragmented lead particles from shot. Dogs frequently consuming such high-lead food risk adverse health effects; this should be considered within decision-making processes about regulation.

**Supplementary Information:**

The online version contains supplementary material available at 10.1007/s13280-023-01856-x.

## Introduction

Lead has a wide range of toxic effects in animals and no blood lead (PbB) concentration has been identified which is considered to be safe for children (CDC 2022). Although most uses of lead have been phased-out or regulated in high-income countries, it remains in common use globally as the principal element in shotgun pellets (shot) and bullets. While passing through the tissues of game animals, lead ammunition frequently leaves behind numerous embedded lead particles of varying size resulting in elevated lead concentrations in meat (Livsmedelsverket 2014; Green et al. 2022a; Pain et al. 2022). Consumption of such meat poses health risks to humans, wild birds and other animals (Kanstrup et al. 2019) and is associated with suppressed population levels and growth rates in raptors in the USA and Europe (Slabe et al. 2022; Green et al. 2022b).

To mitigate the risks posed by lead ammunition, EU and UK authorities are considering banning lead-based ammunition use under Chemicals Regulations (UK and EU REACH Regulations). Evidence reviews conducted to inform these processes (ECHA 2021; HSE 2022) revealed few published studies investigating risks to wild or captive mammals, livestock or domestic animals. Nonetheless, clear exposure pathways to ammunition-derived lead exist, and cases of poisoning have been reported in captive animals and domestic stock (Payne et al. 2013; Kilgallon et al. 2014; Chiverton et al. 2022).

Hunters’ dogs may be fed trimmings from around wound channels and bullet tracts. Animals frequently fed such meat have been assessed as being at high risk from lead exposure (Høgåsen et al. 2016; Knutsen et al. 2019). In a field situation, such dogs have been reported with an arithmetic mean PbB concentration of 32.96 µg/dL (Fernández et al. 2021), approaching the 40 µg/dL threshold suggested as a marker for lead poisoning in dogs (Høgåsen et al. 2016). Hampton et al. (2023) found that a higher proportion of hunter’s dogs had elevated blood lead concentrations (defined as > 1.2 μg/dL in this study) during a deer hunting season, when they are generally fed venison scraps, than prior to it. In one Finnish study, domestic dogs that consumed wild game monthly, weekly, or daily (*n* = 6) had significantly higher PbB than dogs that never consumed wild game (*n* = 29) (Rosendahl et al. 2022).

Other than in hunters’ dogs and this small Finnish study, wider risks to domestic pets have not been explored. However, the feeding of pets with raw meat is increasing (Waters 2017; Dodd et al. 2020), and as this can include game meat, these risks may have increased. Raw meat is now widely available commercially as a main or supplementary petfood, usually supplied as frozen minced meat. While the majority of raw petfood comprises meat such as beef, chicken and pork from domesticated animals, meat from game animals is also used. Products can include minced whole animals, such as gamebirds or rabbits, or parts thereof, which may include offal, bone, fur or feathers.

While gamebird shooting is widespread globally, it is particularly popular in the UK where an estimated 47 million pheasants *Phasianus colchicus* and 10 million red-legged partridges *Alectoris rufa* were released for shooting in 2016, with 15 million and 4.6 million respectively shot (Aebischer 2019). Most gamebirds are shot with lead shot (Green et al. 2022c). Some then enter the human food chain and others the petfood trade, including as raw food. Raw petfood is often minced and during this process lead shot and fragments present in the meat may become further fragmented, increasing their surface area and the potential for gastrointestinal lead absorption.

We investigated the availability of pheasant and other wild game-based raw petfoods online in the UK and analysed lead concentrations in three pheasant-based raw dogfood products and one raw chicken-based product. We also analysed lead in a dried pheasant and partridge product, a dried chicken product, a processed tinned pheasant and goose-based product and a similar chicken product. We compared lead concentrations in the raw pheasant-based dogfood products with previously published data on lead concentrations in pheasant meat marketed for human consumption over a similar time period.

## Materials and Methods

Standard methods were used for literature review, chemical and statistical analysis. Full details and references are provided in Appendix S1.

### Identifying raw petfood products containing game

We used the search engine Google to find UK online suppliers of raw petfood products. For the first 50 suppliers found using the terms ‘Raw, pet, dog, cat, food, UK’ we identified those that sold pheasants and other game animals, whether these appeared to be farmed, wild or potentially wild, and whether they were listed as potentially containing shot.

### Product acquisition and analysis

Petfood may be listed as ‘complete’, i.e., sufficient for a daily ration, or classified as animal feed or ‘complementary’ feed, which are assumed sufficient for a daily ration only if used in combination with other feed. Complete feed has a Maximum Residue Level (MRL) for lead of 5 ppm w.w., assuming a moisture content of 12%, compared with 10 ppm w.w. for general animal feed or complementary feed (EC 2002). We have therefore specifically identified those products purchased as complete animal foods. We purchased the following dogfood products:

#### *Raw* (4 products)

One pheasant-based complete food product (30 packages, one supplier); two pheasant-based products (30 packages from each of two suppliers); one chicken-based complete food product (12 packages from one of the aforementioned suppliers). Descriptions of all three raw pheasant-based products mentioned that they may contain shot, but the shot type was unspecified.

#### *Air-dried* (2 products)

30 packages of air-dried pheasant and partridge sticks and 11 packages of air-dried chicken sticks from one supplier.

#### *Processed wet food* (2 products)

30 tins of complete food derived from a mixture of pheasant and goose carcasses (40% pheasant) and 12 pouches of primarily chicken-based complete food from one supplier.

Product compositions and processing details are given in Table 1 and Appendix S1.

**Table 1:** Numbers of shot and shot fragments and mean lead concentrations in samples from dogfood products containing pheasant sourced in the UK. Mean lead concentrations with different superscript letters (a or b) were significantly different from each other (*P* < 0.05). Products which were labelled as complete feed (rather than general animal feed or complementary feed) are indicated by an asterisk in the product content column. Standard errors of lead concentration could not be calculated for products for which all samples had concentrations below the LOD ($)

Product content (as described at purchase)	Code (Number of packages sampled)	Mean number of shot or fragments observed on X-ray (range) and percent containing shot or fragments			Mean lead concentrations ppm d.w. (SE)	% samples exceeding the maximum residue level (MRL); 11.36 ppm d.w. for animal feed and complementary feed; 5.68 ppm d.w. for complete feed
		Whole shot	Large fragments	Small fragments	Total	
Raw: minced pheasant carcass containing up to 40% bone	PM1 (30)	2.73 (0–6) 93.3%	0.37 (0–2) 33.3%	2 (0–7) 86.7%	551.23^a^ (322.34)	66.7
Raw: 100% minced wild pheasant	PM2 (30)	2.27 (0–7) 80.0%	0.17 (0–1) 16.7%	1.5 (0–5) 73.3%	1 537.50^a^ (689.79)	63.3
Raw: 90% minced pheasant meat containing ca. 10% ground bone, plus 9% vegetable material and 1% oil*	PM3 (30)	0.73 (0–5) 46.7%	0.67 (0–4) 43.3%	4.8 (1–12) 100%	1 391.03^a^ (852.60)	100*
All raw pheasant products combined	(90)	1.91 (0–7)	0.40 (0–4)	2.78 (0–12)	1 159.92 (379.52)	76.7
All raw pheasant products combined excluding samples containing shot ^$^weighted mean – see text	(81)				220.99^$^ (127.14)	74.1
100% pheasant and partridge air-dried sticks	PS (30)	0	0.03 (0–1) 3.3%	1.63 (0–5) 73.3%	30.79^a^ (8.98)	60.0
Processed tinned food. Pheasant 40%, goose 40%, vegetable material ca. 18%, oil 1.5%*	PT (30)	0	0	0.07 (0–1) 6.7%	0.65^b^ (0.03)	0*
Raw fine minced 79.6% chicken (including approx. 10% bone) with vegetables and 1% oil*	CM (12)	0	0	0	0.09^b^ ($)	0*
Processed free range chicken in pouches. 80% chicken plus vegetables*	CP (12)	0	0	0	0.46^b^ (0.03)	0*
Air-dried sticks 70% chicken plus beef meal and minerals	CS (11)	0	0	0.7 (1–3) 54.5%	0.15^b^ ($)	0

### X-ray

**Fig. 1:**
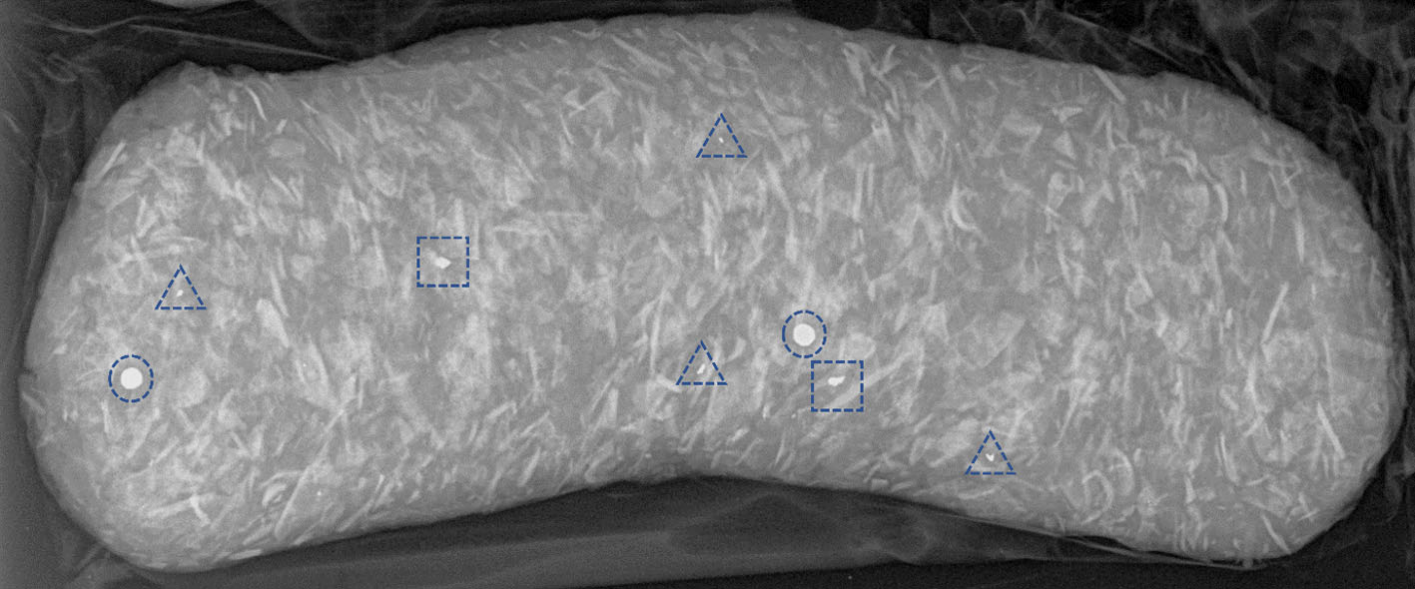
X-ray of a pack of petfood consisting of raw mince derived principally from pheasants. Spherical radio-dense objects assumed to be whole shot are indicated by circles (*n* = 2). Large radio-dense fragments are indicated by squares (*n* = 2) and small fragments by triangles (*n* = 4)

We conducted a two-dimensional X-ray on the whole of each product pack and counted radio-dense objects in three categories: approximately spherical and probably shot; ≥ 0.5 mm diameter irregularly shaped fragments; < 0.5 mm diameter fragments, as illustrated in Fig. 1. Experiments in which lead spheres and bone fragments of similar size were injected into chicken carcasses (Green et al. 2022a) showed that radio-dense metallic particles can be distinguished from bone particles on X-rays because metallic particles are brighter and contrast more with the surrounding tissue (Green et al. 2022a). However, this may become more difficult with smaller particles. While radio-dense particles may comprise metals other than lead, we consider this unlikely in our study as > 99% of pheasants shot and sampled in the UK over our project period (2020–2021 and 2021–2022 pheasant shooting seasons) were shot using lead ammunition (Green et al. 2022c).

While we attempted to remove visible whole shot from all analysed samples, some samples had extremely elevated lead concentrations (see below). We therefore considered the possibility that some shot remained in these samples. We checked this by repeating X-rays of meat remaining in packages from which samples had the 14 highest lead concentrations, and 12 other random packages from the remaining 76.

### Chemical analysis

Following first X-ray, we collected six subsamples, each of ca. 5 g wet weight (w.w.), at random from different locations in the package and pooled them to give a single sample of ∼30 g for each of the packages of wet products tested. Samples were examined macroscopically to remove whole shot. Samples were weighed, dried to constant mass and milled. Complete air-dried products were dried to constant mass before taking a sample for milling. From each milled sample, 0.4 g was digested in nitric acid and samples, certified reference material and blanks were analysed using an inductively coupled plasma optical emission spectrometer (ICP-OES; Agilent 5900). The limit of detection (LOD) for the method for lead was 0.180 ppm dry weight (d.w.) and 0.058 ppm w.w.

Lead concentrations are given as d.w. unless otherwise stated. For raw pheasant dogfood products, on average 1 ppm w.w. = 2.76 ppm d.w.

### Statistical analysis

We used sample lead concentrations to: (1) model the probability distributions of lead concentration among samples from different packs of the same product; (2) compare these distributions among product types and (3) examine the relationship between the probability distributions of lead concentration and the prevalence of radio-dense objects in the products from which the samples were taken. Because data for some products were not normally distributed, we used Kruskall-Wallis one-way analysis of variance by ranks to test for variation in lead concentration among the eight products. We modelled the probability distributions of lead concentration for the samples of each pheasant meat product, assuming a log-normal distribution, and tested their adequacy of fit using the Kolmogorov–Smirnov one-sample test. Where these models did not adequately fit the data for a product at the *P* < 0.05 level, we fitted a mixture model in which sample concentrations were assumed to be bimodal and drawn from two log-normal distributions. We assessed the adequacy of fit, as described above. We tested the correlation between the prevalence of radio-dense objects per unit dry weight and lead concentration using the Spearman rank correlation coefficient on the means for the eight products. Statistics followed Siegel and Castellan (1988). Means presented throughout the text are arithmetic.

### Lead concentrations in pheasant meat marketed for human consumption

As part of a project reported on by Wild Justice (2021), 75 pheasant products (breast meat or whole birds) marketed for human consumption were purchased from three retailers in the UK between January and November 2021, within the 2020–2021 and 2021–2022 pheasant shooting seasons. Subsamples of muscle tissue were taken and analysed for lead as described in SI Appendix 2 of Pain et al. (2022). The analysis was undertaken at the Environmental Research Institute under the supervision of one of the authors of the current paper (MAT); results are directly comparable with those of dogfood presented here. We compared lead concentrations in the meat of pheasant products marketed for human consumption and those sold as raw dogfood. The sample collection time periods overlap and cover two pheasant shooting seasons in which > 99% of pheasants sampled as part of a UK monitoring programme were found to have been shot with lead ammunition (Green et al. 2022c). To facilitate comparison of the distributions of concentrations in the lower part of the range, we excluded samples in which lead shot had been found to be present at analysis.

## Results

### Availability of raw petfood containing game

Of 50 online suppliers surveyed, 8% sold only cat food, the remainder sold dogfood or both. Wild game was sold by 46% of suppliers and potentially wild game (origin unspecified) by a further 22%. Raw minced pheasant (assumed wild-shot) was sold by 34% of suppliers; 71% of these stated that the meat might contain shot, although the shot type (lead or other) was seldom specified. The composition of minced pheasant products varied from primarily meat to also including ground bone or whole minced birds.

### Lead concentrations in dogfood

Lead concentration varied significantly among the eight products (KW = 132.75, *P* < 0.0001; Table 1). Multiple-comparison pairwise Kruskall-Wallis tests identified two groups: a high concentration group comprising raw (PM1, PM2, PM3) and dried (PS) pheasant products; a low concentration group comprising the chicken (CM, CP, CS) and processed pheasant (PT) products. No significant pairwise differences existed within these two groups, but all differences between products in different groups were significant (Table 1).

Radio-dense particles resembling whole shot and/or large fragments were observed in raw pheasant products and one dried pheasant product. Small radio-dense fragments were observed in all products except raw and processed chicken products (Table 1; Table S1), but their identification became increasingly subjective as size decreased. Variation among the eight products in the mean lead concentration tended to be positively correlated with the mean number of radio-dense objects per unit dry mass, though the correlation was not significant for small fragments (*r*_*s*_ = 0.791, two-tailed *P* = 0.019; *r*_*s*_ = 0.786, *P* = 0.021; *r*_*s*_ = 0.455, *P* = 0.257 for shot, large fragments and small fragments, respectively).

Probability distributions of lead concentration from dried pheasant sticks and processed tinned dogfood conformed adequately to the one-group log-normal model (Table S2). For the three raw pheasant products (Table S2), the one-group models did not give an adequate fit (Kolmogorov–Smirnov one-sample tests; *P* < 0.05) but the two-group log-normal models did (*P* > 0.20). These products had bimodal distributions of lead concentration (Fig. S1), with the high concentration group having a mean concentration 198 to 694 times higher than the low concentration group (Table S2).

**Fig. 2:**
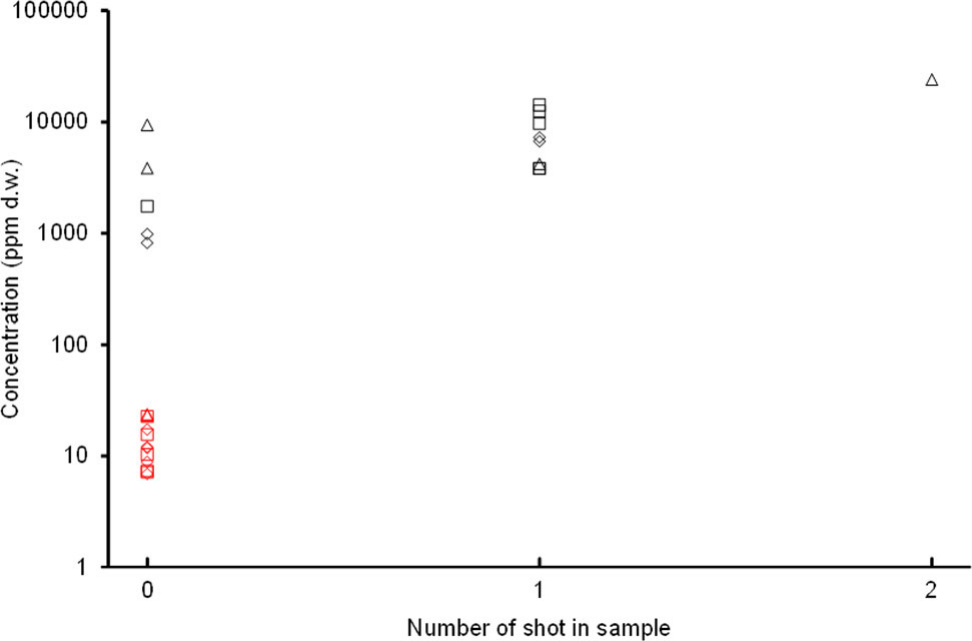
Concentrations of lead (ppm d.w.) in meat samples taken from packages of three raw-minced pheasant dogfood products in relation to the number of whole shot inadvertently included within the meat sample when it was taken from the package and dried/milled. Legend: Results are shown for two sampling strata: all 14 of the packages with the highest lead concentrations in their meat samples (black symbols) and a random sample of 12 packages drawn from the 76 packages with lower concentrations (red symbols). The three product types can be identified from the symbol shape (diamonds = PM1, squares = PM3, triangles = PM3). Lead concentration was significantly positively correlated with the number of shot present within the meat sample across the 14 samples with the highest concentrations (Spearman rank correlation coefficient *r*_*S*_ = 0.651; two-tailed *P* = 0.012). The arithmetic mean lead concentration in all 14 samples was 7 351.60 ppm d.w. and in the five samples with no shot from the high concentration stratum was 3 368.59 ppm d.w

Repeat X-ray of minced pheasant packages (products PM1, PM2, PM3) from which samples had the 14 highest lead concentrations showed that two shot remained in one milled sample and a single shot remained in eight samples. The remaining five samples contained no shot, nor did any of the 12 samples selected from the 76 packages in the lower lead concentration group (Fig. 2). The mean lead concentration in the five high-lead samples with no shot was 3 368.59 ppm d.w. The 12 samples randomly selected from the lower concentration group had a mean lead concentration of 13.92 ppm d.w. We assumed that all 76 low-concentration samples contained no shot and had the same mean lead concentration. The weighted mean for all samples known or assumed to contain no lead shot was therefore (76 × 13.92 + 5 × 3 368.59)/81, i.e., 220.99 ppm d.w. with 95% confidence limits of 78.84 – 421.14 ppm d.w., calculated using a bootstrap procedure (Appendix S2). We conclude that 10% (9/90) of the minced pheasant samples had very much higher lead concentrations than the remaining 90% of samples because of the presence of lead shot in the sample.

The EU MRLs for lead in animal feed/complementary feed and in complete feed are 10 and 5 ppm w.w., respectively, assuming a moisture content of 12% (EC 2002; for the UK see https://www.legislation.gov.uk/eudr/2002/32), equivalent to 11.36 and 5.68 ppm d.w., respectively. These MRLs were exceeded in samples from > 60% of two raw pheasant animal feed products (PM1, PM2), all samples of the raw pheasant complete feed product (PM3) and 60% of samples of the dried pheasant/partridge sticks. When excluding samples containing whole shot, 74% of the raw minced pheasant product samples combined exceeded these MRLs. No samples from other products analysed exceeded the MRL (Table 1).

### Comparison of lead concentrations in raw pheasant food marketed for humans and dogs

**Fig. 3:**
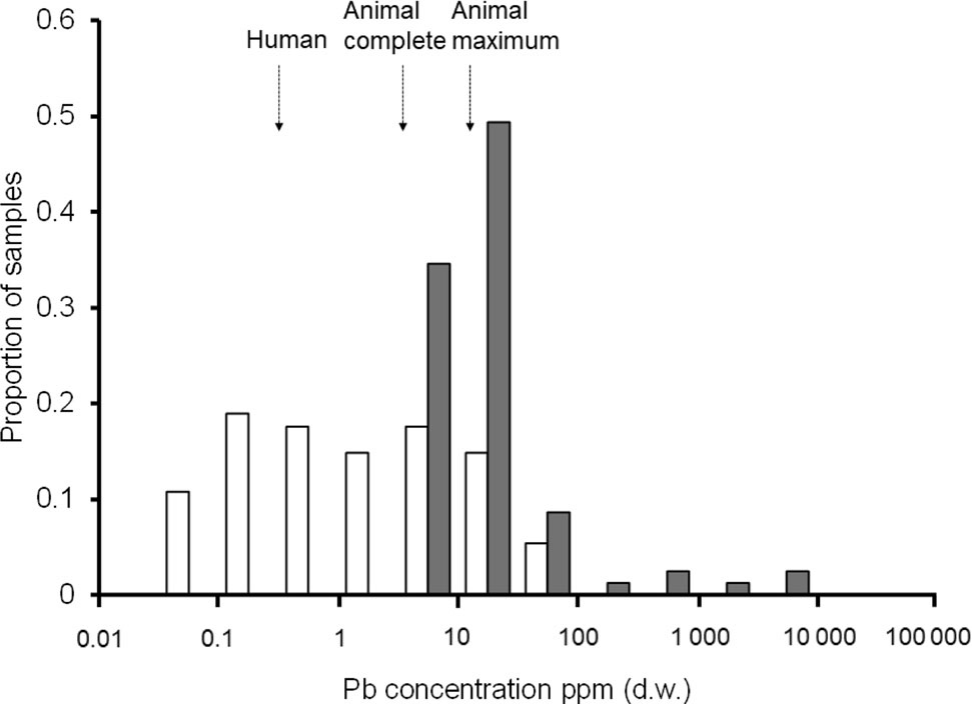
Distribution of concentrations of lead (ppm d.w.) in pheasants sold in shops for human consumption from three UK food retailers in 2021 (white bars; *n* = 74, mean = 6.46 ppm d.w.; Wild Justice 2021) and from three online retailers of raw minced pheasant dogfood in 2022 (grey bars; *n* = 81, mean = 220.99 ppm d.w.; this study). Measurements are binned by equal categories of log_10_ concentration (two categories per order of magnitude). Arrows indicate the EU MRL for meat from domesticated animals for human consumption (0.1 ppm w.w., converted to 0.307 ppm d.w., EC 2006), the MRL for complete feed (5.68 ppm d.w.) and the maximum MRL for animal feed/complementary feed (11.36 ppm d.w.). Samples inadvertently containing shot (when milled, digested and analysed) were excluded from both datasets

The distribution of lead concentrations in meat from pheasants sold in shops for human consumption from three UK food retailers in 2021 (covering the 2020–2021 and 2021–2022 pheasant shooting seasons), and from raw minced pheasant dogfood from three online retailers in 2022 (during the 2021–2022 pheasant shooting season) is presented in Fig. 3. Raw data are available in Table S3.

As reported in Pain et al. (2022), 69% (51/74) of pheasant meat samples sold for human consumption exceeded the EU MRL of 0.100 ppm w.w. set under EU Regulation1881/2006 (EC 2006) for lead in muscle tissue of domestic animals (poultry, pigs, sheep and cattle) destined for human consumption. While no formal level has been set for game meat under this regulation, EU and UK legislation requires the analysis of samples from food producing animals for contaminant residues, including lead. This involves an annual surveillance plan which is operated by the Veterinary Medicines Directorate in the UK. The reference point used for lead in the meat (muscle tissue) of domestic stock and wild game animals is 0.100 ppm w.w., equal to the EU MRL (VMD 2014). Samples with a lead concentration exceeding this are considered to be non-compliant. In the UK, residues surveillance is covered by The Animals and Animal Products (Examination for Residues and Maximum Residue Limits) Regulations (https://www.gov.uk/guidance/residues-surveillance).

Excluding samples in which shot were found, the weighted mean lead concentration in raw pheasant dogfood samples (220.99 ppm d.w) was 34 times higher than the mean lead concentration in meat destined for human consumption (6.46 ppm. d.w.), with relatively little overlap in the two distributions (Fig. 3). No raw dogfood pheasant products had lead concentrations below the EU MRL for meats destined for human consumption (0.100 ppm w.w. ≅ 0.307 ppm d.w.).

## Discussion

### Lead concentrations in dogfood

When analysing risks from dietary exposure to lead of ammunition origin, the inclusion or exclusion of samples with very elevated concentrations, so called ‘outliers’, is debated because they probably result from whole shot or large fragments of lead, less of which is likely to be dissolved in the intestine and absorbed than for smaller particles. However, as an unknown proportion of larger lead particles may be absorbed, and understanding of the physiological and dietary conditions that influence absorption is limited, complete lead distributions are sometimes used for risk assessment (e.g., ECHA 2021). Additionally, Pain et al. (2022), in a multi-country analysis of lead concentrations in the meat of small game animals, found that variation among means for individual studies declined markedly with increasing numbers of carcasses sampled and was approximately symmetrical on a logarithmic scale about the geometric mean at all sample sizes, suggesting that extreme values were probably attributable to small sample size. In raw minced pheasant dogfood, we found that 10% of small samples taken from the packages had high lead concentrations because of the presence of whole shot in the sample. The remaining 90% of samples contained no shot and had a lower, but still high, weighted mean lead concentration of 220.00 ppm d.w. (Fig. 2; Table 1). This finding supports the inclusion of both the high- and low-concentration samples for the evaluation of potential risks.

Our results indicate that the pheasants used in the three raw and one dried dogfood product analysed were mainly killed with lead shot. Lead concentrations exceeded the EU MRL for animal feed/complete feed in samples from approximately three quarters of raw pheasant-based dogfood packs from three products. We are not aware of previous lead analysis in raw pheasant-based petfood. Mean lead concentrations previously reported in commercially available dry and wet (tinned) complete petfood range from well below (e.g., Paulelli et al. 2018; 0.29 ppm d.w.), to above (e.g., Zafalon et al. 2021; 12.55 ppm d.w.), the EU MRL. However, the maximum concentration found by Zafalon et al. (2021) was 21.82 ppm d.w., an order of magnitude lower than the mean concentration in raw pheasant-based dogfood here (Table 1). This is probably because the meat in most commercial petfood previously analysed was from domestic stock such as cattle, chickens, pigs, and also from fish, rather than wild-shot game. We found that one processed (tinned) dogfood product in our study containing 40% pheasant had a low mean lead concentration (0.65 ppm d.w.; Table 1), possibly relating to the origin of the pheasants and/or processing methods used. For example, some pheasants reared on game farms may have been slaughtered while captive and used for petfood, or may have been released birds sourced from shoots that use non-lead ammunition.

### A comparison of lead concentrations in raw pheasant food marketed for humans and dogs

The mean lead concentration in raw dogfood samples was surprisingly high, exceeding by 34 times that of raw pheasant meat sold in UK retail outlets for human consumption, which itself is 21 times the EU MRL for meat from domesticated animals destined for human consumption (Fig. 3).

We suggest three possible reasons for this. First, raw pheasant dogfood was minced, whereas that sold for human consumption is generally meat from whole breasts or oven-ready (eviscerated) intact birds. Mincing may further fragment lead shot or particles already present, increasing the number of very small lead particles, resulting in a more homogenous distribution and increasing lead concentrations in analysed samples. The higher number of small fragments and concomitant larger total surface area is also likely to increase the potential for gastrointestinal lead absorption by dogs.

Second, some pheasant dogfood contained bone, organs and presumably viscera (Table 1). However, while biologically incorporated lead is disproportionately deposited in bone, kidney and liver, its contribution is likely to be minor compared to lead derived directly from ammunition (Pain et al. 2022); the lowest mean lead concentration was found in the raw pheasant product containing the highest proportion of bone.

Third, Regulation (EC) No 1069/2009 (EC 2009; for the UK see https://www.legislation.gov.uk/eur/2009/1069/) permits operators to place a range of petfood on the market from ‘Category 3’ material, including animal by-products, which, in the case of game, includes the bodies or parts of game animals killed, which are fit for human consumption in accordance with Community legislation, but are not intended for human consumption for commercial reasons. Thus, it appears possible that game animals surplus to requirements for human consumption or perhaps badly/obviously damaged by ammunition may enter the raw petfood market. Tissue may be obviously damaged if hit by a larger number of shot, where shot has hit bones, or for other reasons. Such tissue could contain more shot and/or ammunition fragments, and thus have higher lead concentrations (Pain et al. 2010 and this study).

### Potential health risks to dogs consuming raw pheasant dogfood

Most lead poisoning in dogs results from ingestion (Berny et al. 2012) and lead particularly affects the gastrointestinal, nervous, haematological, renal and cardiovascular systems (Høgåsen et al. 2016; Bates 2018). Increases in PbB concentrations have been associated with ingestion of ammunition-derived lead in correlative studies of humans (Green & Pain 2012) and dogs (Fernández et al. 2021; Rosendahl et al. 2022) and in an experimental study in pigs (Hunt et al. 2009).

Gastrointestinal absorption of lead ions solubilized from ingested metallic lead may be lower than absorption of lead from some other dietary sources, and is associated with particle size and surface area. For example, Barltrop & Meek (1979) found an ~ fivefold increase in metallic lead absorption in rats fed metallic lead particles of 6 µm relative to 197 µm. Green et al. (2022a) imaged eight pheasants in three dimensions using a micro-CT scanner and found that 79% of 340 metallic fragments identified were 70–300 µm in diameter, with 37% < 100 µm in diameter. Particles < 70 µm could not be detected but may have been present. Tens of millions of nanoparticles per gram of meat, down to a detectable size of approximately 50 nm, have been found in the wound channels of large mammals shot with lead bullets (Kollander et al. 2017). However, this has not been investigated in the tissues of animals killed with lead shot, the ballistic properties of which are different to those of bullets. The increased potential for gastrointestinal absorption of lead from small metallic particles has particular relevance for the current study, as lead fragments in the meat of shot pheasants may be further fragmented by the mechanical mincing process used to prepare minced pheasant dogfood.

Høgåsen et al. (2016) assessed the health risks to dogs fed trimmings of lead-shot game, assuming a wide range of gastrointestinal absorption (10–80% of that of lead acetate) to reflect the variability in particle size and uncertainty about the bioavailability of metallic lead in dogs. These authors considered that dogs frequently fed trimmings of lead-shot game may suffer adverse health effects from exposure to the amounts of lead present, and that even lethal exposure could occasionally occur.

Raw meat feeding websites suggest that suitable amounts of complete raw food for dogs range from about 10% of body weight/day for a young puppy (2–4 months) to about 2.5% of ideal body weight/day for an adult dog. Assuming a feeding range of 25-100 g meat/kg b.w/day for animals being fed exclusively raw food, and a mean lead concentration of 504 ppm w.w. (1 391 ppm d.w. for product PM3, which was the only minced pheasant product labelled as a complete food; Table 1), this represents a lead intake of 12.6–50.4 mg Pb/kg b.w./day, corresponding to a range of 1.26–40.32 mg Pb/kg b.w./day of lead acetate equivalents (as per Høgåsen et al. 2016). Even assuming a 10% absorption rate of ammunition-derived lead relative to lead acetate this dose exceeds the Lowest Observed Effect Level (LOEL) suggested by Høgåsen et al. (2016) of 1 mg/kg b.w./day. This suggests that dogs of all ages frequently fed raw pheasant meat containing the average concentration in the complete raw pheasant food purchased here may be at risk from adverse chronic health effects of varying severity.

A lowest lethal dose of 300 mg lead acetate/kg b.w. has been proposed for dogs (CDC 1994) representing 375–3 000 mg of ammunition-derived lead/kg b.w. as per Høgåsen et al. (2016). Three of our 90 samples (3.3%) of raw dogfood had lead concentrations exceeding 3 750 ppm w.w., (4 442 – 8 550 ppm w.w.). This suggests that for young puppies with higher food intake rates, and assuming higher levels of lead absorption, the possibility of occasional lethal exposure cannot be discounted. These results are broadly similar to those of the deterministic risk assessment conducted by Høgåsen et al. (2016) for dogs fed trimmings from wound channels of large game animals.

### Reducing lead poisoning risks

The risks presented by ammunition-derived lead to the health of humans, wildlife, domestic pets and other animals that consume wild game could be quickly and effectively eliminated through the use of non-toxic ammunition, which has long been available. For example, Denmark banned the sale, possession and use of lead shot for all shooting in 1996. Compliance with this ban is high and has proved effective in reducing lead concentrations in wild game meat, and thus associated risks to human and wildlife health (Kanstrup & Balsby 2019; Pain et al. 2022). In 2022 Denmark also announced that lead rifle bullets will be banned for hunting (https://ww.w.retsinformation.dk/eli/lta/2022/971), making it the first country to ban all lead ammunition for hunting.

In the UK, concern over this issue stimulated some retail outlets to pledge to sell wild game (marketed for human consumption) killed only with non-lead ammunition (LAG 2022). Additionally, nine UK shooting and rural organisations announced in February 2020 their intention to encourage voluntary transition to non-lead shotgun ammunition for hunting within five years (BASC 2020). However, research in the 2020/21 and 2021/22 pheasant shooting seasons found that > 99% of pheasants were still being killed with lead shot (Green et al. 2022c). Both voluntary approaches, and partial regulations, i.e., banning the use of lead shot only for shooting wildfowl and/or over wetlands, have been shown to elicit poor compliance in the UK and elsewhere in Europe (Cromie et al. 2015; Pain et al. 2022), in contrast with the complete and successful ban on lead shot in Denmark.

Lead ammunition bans proposed under both UK and EU REACH Regulations are being scrutinized to ensure that key risks have been identified and quantified, and that proposed mitigation measures are suitable and proportionate, given their associated cost/benefit. Risks from lead ammunition to the health and welfare of pets fed raw food containing wild-shot game should be adequately considered in these processes.

## Conclusion

About three quarters of raw pheasant dogfood samples analysed had lead concentrations exceeding maximum levels permitted in animal feed, which are ∼19–37 (for complete and other feedstuffs) times higher than those set for meat for human consumption. As lead affects all mammals in a broadly similar way, and some dogs and other carnivorous mammals might be fed almost exclusively on pheasant meat, it may be appropriate to review the permitted maximum lead levels in animal feed.

Lead concentrations in raw pheasant dogfood possibly exceeded those in raw pheasant marketed for human consumption due to additional fragmentation of lead from shot during mechanical mincing of carcasses in the processing of raw petfood. Frequent exposure of dogs to such dietary lead levels is likely to present health risks. As UK households own an estimated 13 million dogs and 12 million cats (2022; ukpetfood 2023), and raw diets are increasingly popular within and beyond Europe (Dodd et al. 2020), numbers of pets at risk could be high.

To mitigate these risks, petfood suppliers could source wild game killed with non-lead ammunition, and some may already do this. Pet owners could check this when purchasing petfood. This issue could also be addressed through enhanced monitoring and enforcement of existing regulations on undesirable substances in animal feed (EC 2002), but this would be costly and would not protect the dogs of hunters or pet owners that prepare petfood including wild game at home. An overarching One Health approach of replacing lead with non-toxic ammunition, as is currently being considered under UK and EU REACH regulatory processes, would remove these risks while also benefitting humans, wildlife and the environment.

## Supplementary Information

Below is the link to the electronic supplementary material.

Supplementary file1 (PDF 313 kb)
