# Supplementary material for: Lead concentrations in commercial dogfood containing pheasant in the UK
Source: Ambio. 2023 May 3;52(8):1339–49. doi: 10.1007/s13280-023-01856-x (PMC10272007; doi:10.1007/s13280-023-01856-x)
Supplement: Supplementary file 1 — Supplementary file1 (PDF 313 kb) [file 13280_2023_1856_MOESM1_ESM.pdf]

Ambio
Electronic Supplementary Material
This supplementary material has not been peer reviewed.

Title: **Lead concentrations in commercial dogfood containing pheasant in the UK**

Authors: Deborah J. Pain, Rhys E. Green, Nicola Bates, Maider Guiu, Mark A. Taggart

Appendix S1 - Methods

Identifying raw petfood products containing meat from wild-shot game animals: We used the search engine Google to find UK online suppliers of raw pet food products. We searched for the first 50 listed suppliers of raw petfood using the terms 'Raw, pet, dog, cat, food, UK'. We recorded whether each product offered for sale was described as having been derived from game animals or not, and whether the game animals were described as wild or not. We considered the following types of game animal specified by suppliers to be potentially wild game: deer (venison), rabbit, hare, pheasant, partridge, goose and pigeon. Ducks are commonly farmed with much smaller numbers shot in the wild, so we did not consider duck products to be likely to be derived from wild game, although it is possible that some wild shot duck may enter the petfood trade. Pheasants and partridges are commonly reared on game farms in the UK for release to the wild for shooting. In 2016, it is estimated that approximately 57 million pheasants and partridges were released to the wild with approximately 20 million of these subsequently shot in the same year (Aebischer 2019). We therefore assume that the majority of pheasant and partridge carcasses used for pet food in the UK are likely to have been wild-shot and not to have been slaughtered at farms or abattoirs. Our understanding is that hares are rarely captive-bred for food in the UK and are also all likely to have been wild-shot. We thus classified pheasants, partridges and hares sold in the raw petfood trade as wild game. The remaining animals (deer, rabbit, goose and pigeon) may either be shot in the wild or farmed before being slaughtered. Some suppliers stated that their product contained wild rabbit, others farmed rabbit, and some suppliers used both. We thus considered all of these species to be potentially wild game, unless their origins were stated. For products stated to contain meat from pheasants, we searched the website for any statement suggesting that the product might contain shotgun pellets (shot).

Sample acquisition and analysis: We purchased 30 packages of each of three different dogfood products containing frozen raw pheasant from three suppliers. We also purchased 30 packs of air-dried pheasant and partridge sticks (one product), and 30 tins of processed wet food derived from a mixture of pheasant and goose carcasses (one product, 40% pheasant) from one supplier each. We chose products for which the suppliers specified the composition of the food and the proportion of pheasant it contained. The exception was air-dried sticks where the proportions of pheasant and partridge were not specified. However, both types of gamebirds were likely to have been wild-shot (see above).

We also purchased dogfood derived principally from domestic fowl (chicken) carcasses for comparison with those containing pheasant. We purchased 12 packages of raw food, 12 pouches of one type of processed wet food and 11 packs of one brand of air-dried sticks. These were purchased from suppliers from which we also obtained the pet food containing pheasant.

On receipt at the laboratory products were removed from their original packaging, placed into large zip-lock sample bags and labelled with a unique sample code. During this process, relevant product information was recorded including details regarding manufacturer, batch number, product pack size, main listed product ingredients and production date.

X-ray: We conducted two-dimensional X-ray on the whole of each product pack. The X-ray generator used was a Medical Econet meX+40, with generator settings of 50kV and 2mAs. The processor used was an Agfa CR30-X. On the X-ray of each pack, we counted the number of approximately spherical radio-dense objects, which contrasted strongly with the background. These are likely to have been whole or almost whole shot. We also observed smaller radio-dense objects with irregular shapes, which were likely to have been fragments of metal. We counted numbers of these in two categories, large and small, based upon a subjective assessment of mean diameter in the plane of the image. The

large fragments were approximately $\geq$0.5mm diameter, based upon a comparison of their size with that of the large spherical objects. In making this estimate, we assumed that the spherical radio-dense objects were probably a mixture of #5 (diameter 2.8 mm) and #6 (2.6 mm) shot, because these types are commonly used for shooting pheasants and partridges in the UK. We observed some smaller objects (approximately <0.5mm diameter) which also contrasted with the background, but we were uncertain whether these were all metal fragments. Because of this our counts of small objects were somewhat subjective and probably represent minimum numbers of metal fragments.

Preparation for chemical analysis: After X-ray was completed, we took a sample from each package and placed it in a disposable petri dish. The wet mass of the single sample taken from each package was approximately 30g for all wet products tested. The sample was collected by flattening the entire product mass within the zip-lock bag, then using a stainless-steel spatula to collect six large (approx. 5g) subsamples at random from different locations across the package contents and pooling these onto the petri dish to obtain a representative sample. Care was taken to seek to avoid, as far as possible, including any whole shot. This subsample was then dried to constant mass in a drying oven at 65°C, which resulted in a dry weight for each sample. For the air-dried sticks, contents of the whole pack were dried to constant mass before taking a sample for the determination of lead concentration. The moisture content in the pheasant sticks was found to be negligible and to be about 4% in the chicken sticks. Accurate mass measurements and moisture contents were recorded for all samples to allow conversion between wet and dry weight lead concentration values and calculation of the dry mass of the samples and the proportion of the package dry mass taken for the determination of lead concentrations. Having dried the samples, the whole of each was then milled to a fine powder using a food grade spice/coffee mill. Milled samples were then transferred and stored in zip-lock bags in a desiccator post mill to ensure they remained dry prior to analysis. The mill was cleaned thoroughly between samples using dry lab tissue and a stream of compressed air to remove all residual particles of material.

Determination of lead concentration: A 0.4g aliquot of each powdered sample was taken and digested in a pressurised Teflon vessel using a microwave digestion system (Anton Paar Multiwave Pro). Trace metal grade nitric acid and trace metal grade hydrogen peroxide were used for the digestion. Following complete dissolution, the solutions were made up to 15ml final volume with Type I Milli-Q ultrapure water. In parallel with samples, blanks were run to attain a procedural limit of detection for the process, and a certified reference material (CRM) was used to ensure Pb recovery was within tolerance (strawberry leaf powder (LGC 7162) was used as the CRM, with a certified Pb level of 1.8 +/- 0.4 ppm d.w.). Once digestions were complete, all samples/CRMs/blanks were analysed using an inductively coupled plasma optical emission spectrometer (ICP-OES) system (Agilent 5900). The instrument was calibrated against five Pb wavelengths and the 220.353 nm Pb line was used for final data processing/calculations. CRM recovery was within the above CRM tolerance (1.54 +/- 0.26 ppm d.w.; n = 14) and no data correction was applied for recovery. The limit of detection (LOD) for the method for lead was 0.180 ppm d.w. and 0.058 ppm w.w.

Statistical analysis: The objectives of our analysis were (1) to make a quantitative statistical model of the probability distributions of lead concentration among samples from different packs of the same product, (2) to compare these distributions among product types and (3) to examine the relationship between the probability distributions of lead concentration and the prevalence of radio-dense objects in the products from which the samples were taken.

We tested for variation in lead concentration among the eight products examined using Kruskall-Wallis one-way analysis of variance by ranks (Siegel & Castellan 1988). We chose this nonparametric method because preliminary examination of the data indicated that sample-specific concentration values for

some products did not follow either a single normal or single log-normal distribution. We first tested for variation among all of the products and then, having found significant variation, we performed tests in which all pairs of products were compared. For these pairwise tests, we adjusted for multiple testing using the multiple-comparisons method described by Siegel & Castellan (1988).

We next made a statistical model of the probability distribution of lead concentration for the samples of each of the five products derived from pheasant meat using a two-stage procedure. In the first stage, we $\log_e$-transformed the concentration values and calculated the mean and standard deviation for each product. There were two products derived from domestic fowl (CM and CS – product labels given in Table S1) for which there were values for some samples (12/12 and 8/11 respectively) below the limit of detection (LOD). We assumed that these samples had concentrations which were half (0.09 ppm d.w.) of the LOD (0.18 ppm d.w.). In the second stage of the analysis, we tested the adequacy of fit of the modelled distributions using the Kolmogorov-Smirnov one-sample test (Siegel & Castellan 1988). If the Kolmogorov-Smirnov maximum discrepancy (D) value for a product was non-significant ($P > 0.05$) we accepted a one-group log-normal model as an adequate description of the distribution. For products with a significantly large D value ($P < 0.05$) we rejected the one-group model and fitted a mixture model in which the concentration values were assumed to be drawn from two log-normal distributions, each with its own mean (M) and standard deviation (S) and with a proportion k of samples belonging to a group with high concentrations and a proportion ($1-k$) from a group with low concentrations. To obtain maximum-likelihood estimates of k and the two M and two S values for each group we used the NONLIN module of SYSTAT (Wilkinson 1990). After fitting the two-group models, we assessed the adequacy of their fit using the Kolmogorov-Smirnov one-sample test, as described above.

To obtain product-specific measures of the prevalence of radio-dense objects, we divided the count per pack of each of the three object types by the dry mass of the product pack. This adjustment was necessary because products varied considerably in size and in their proportion of dry matter (Table S1). We tested the correlation between these measures of prevalence of radio-dense objects and lead concentration using Spearman rank correlation coefficient (Siegel & Castellan 1988) on the means for the eight products.

It seemed possible that the superior fit of the two-group log-normal model to lead concentration data for some products might be accounted for if the sample from some of the packs contained one or more radio-dense shot and/or fragments whilst the other samples from packs of the same product happened by chance not to include any fragments. If the radio-dense fragments were composed of lead, this stochastic variation might account for the difference between the lead concentrations of the high and low-concentration sample groups. To assess this possibility, we calculated the expected mean number m of large and small fragments combined in a sample of each product from the mean numbers of fragments per pack and the dry weight of the sample relative to that of the pack. We took the expected proportion of samples of a product with at least one shot and/or fragment present in it to be likely to be determined by the Poisson distribution and therefore to be given by $1 - e^{-m}$.

Repeat X-ray: Our analysis indicated that samples taken from packages of the three products (PM1, PM2, PM3) derived principally from raw minced pheasant had high arithmetic mean lead concentrations and the probability distribution of concentrations across samples from different packages (one sample per package) showed two peaks. We considered it possible that this bimodal distribution had arisen because the samples with the highest concentrations contained whole shot. We had intended to remove all shot from the samples before analysis so that our concentration measurements would reflect the concentration of lead from lead fragments, which is most likely to be absorbed. Although we did succeed in removing shot from some of the meat samples, it seemed

possible that we had failed to find shot in some of them. To check for this possibility, we repeated the X-ray, as described above, on the meat remaining in some of the packages of products PM1, PM2 and PM3. We did this on a stratified sample of packages, with stratification based upon the measured dry weight concentration of lead in the sample taken from the package. We ranked all of the PM samples from highest to lowest based upon lead concentration and carried out repeat X-ray on the remaining contents of the packages with the 14 highest concentrations. From the remaining 76 packages we conducted repeat X-ray on a set of 12 packages selected at random. The number of shot remaining in each package was counted and subtracted from the number counted previously in the whole package to give the number of shot inadvertently left in the meat sample taken for determination of lead concentration.

Appendix S2 – Results

Lead concentrations in dog food

There was statistically significant variation among the eight products in their concentration of lead (KW = 132.75, $P < 0.0001$; Table S1). Multiple-comparison pairwise Kruskall-Wallis tests indicated that there were two groups of products: a high concentration group consisting of four of the five products derived from pheasant meat (PM1, PM2, PM3 and PS) and a low concentration group consisting of all three of the products derived from domestic fowl and the tinned pheasant and goose product (CM, CP, CS and PT). There were no significant pairwise differences within these two groups, but all differences between products in different groups were significant (Table S1). Variation among the eight products in the arithmetic mean concentration of lead tended to be positively correlated with the mean number of radio-dense objects per unit dry mass, though the correlation was not significant for small fragments ($r_s = 0.791$, two-tailed $P = 0.019$; $r_s = 0.786$, $P = 0.021$; $r_s = 0.455$, $P = 0.257$ for shot, large fragments and small fragments respectively).

The probability distributions of lead concentration of two of the five products derived from pheasant meat (PT and PS) conformed adequately to the one-group log-normal model, according to the Kolmogorov-Smirnov one-sample tests (Table S2). However, the one-group log-normal model did not adequately describe the probability distributions of any of the three raw mince pheasant products (PM1, PM2, PM3; Table S2), whilst the two-group log-normal model gave an adequate description for all three of these products (Kolmogorov-Smirnov one-sample tests; $P > 0.20$ in all cases). Inspection of the distribution data confirmed that the three raw mince pheasant products had bimodal distributions of lead concentration (Figure S1), with the high concentration group having an arithmetic mean concentration much higher than the low concentration group: 198 to 694 times higher (Table S2). We suggest that the high concentration group of (milled) samples might be those which happened to include one or more shot and/or fragments of lead large enough to be visible on our X-rays, with the low concentration group being those with no fragments detectable using the X-ray equipment we used. Comparison of the estimated proportion of high-concentration samples from the raw pheasant mince products (mean 0.168, range 0.133-0.200) with the Poisson-derived estimates of the proportion of samples of these products likely, by chance, to include one or more shot and fragments (mean 0.282, range 0.232-0.308) or one or more fragments of both sizes (mean 0.180, range 0.017-0.277) suggests that this hypothesis may be correct. We compared the proportion of samples in the high group with both of these Poisson probabilities because we did not always exclude whole shot from the samples, as intended (see below). It should be noted that the arithmetic mean concentration of lead even for the low concentration group of samples of the pheasant mince products was still high: ranging for 12.65 to 18.57 ppm d.w. (Table S2). Hence, even the low concentration group of pheasant mince samples had lead concentrations two orders of magnitude higher than those for products derived from the meat of domestic fowl (range 0.09 – 0.46 ppm d.w.; Table S1). The high concentration

of lead in the low-concentration samples, which we hypothesise included no fragments of lead detectable on X-rays, suggests that there were many fragments of lead present that were smaller than the detection threshold for our X-ray equipment.

Repeat X-ray of the 14 packages of PM products with the highest lead concentrations in the meat samples taken from them showed that a single shot had been present in samples taken from 8 packages, two shot in one sample and that the other five samples had not contained any shot. Lead concentration was significantly positively correlated with the number of shot present within the meat sample across the 14 samples with the highest concentrations (Spearman rank correlation coefficient r_S = 0.651; two-tailed $P < 0.05$). The arithmetic mean concentration of lead of all 14 samples was 7 351.60 ppm d.w. and of the five samples with no shot from the high concentration stratum was 3 368.59 ppm d.w.

Shot were absent from the meat samples taken from all of the 12 packages randomly-selected from the 76 packages whose samples had lower concentrations. The arithmetic mean concentration of lead in these samples was 13.92 ppm d.w. We calculated the arithmetic mean lead concentration expected for meat samples from all packages if no shot had been present as the weighted mean of concentrations for samples in packages with no shot in their meat sample in the two strata used for the repeat X-ray. We assumed that all packages in the low concentration stratum had no shot present in them and therefore that the total number of PM packages with no shot in the meat samples was 76+5 = 81. The arithmetic mean lead concentration expected for meat samples with no shot was therefore the weighted mean of 3 368.59 ppm and 13.92 ppm, with weights 5/81 = 0.062 and 76/81 = 0.938. This weighted mean is 220.99 ppm d.w. We calculated the 95% confidence limits of this weighted mean using a bootstrap procedure. We drew 5 concentrations from the observed values in samples with no shot in the high concentration stratum at random, with replacement, and 12 concentrations from the observed values in the lower concentration stratum, also at random and with replacement. We then calculated the arithmetic mean concentrations for the two strata from these values and their weighted mean, as described above. We repeated this procedure 10 000 times, ranked the bootstrap weighted means and took the bounds of the central 9 500 values to be the 95% confidence limits of the weighted mean. This confidence interval was 78.84 – 421.14 ppm d.w.

Table S1. Mean numbers of radio-dense objects recorded on X-rays of the whole package and arithmetic mean concentration of lead in one sample taken from each package. Mean concentrations with different superscript letters (a or b) were significantly different from each other (*P* < 0.05). Standard errors of lead concentration could not be calculated for products for which all samples had concentrations below the LOD ($).

							Mean number per package			Mean number per kg d.w.				
Principal species	Product type	Short name	# Packages	% dry weight	Mean package dry weight (g)	Sample dry weight (g)	Whole shot	Large Fragment	Small Fragment	Whole shot	Large Fragment	Small Fragment	Mean Pb ppm d.w.	SE
Pheasant	Raw mince	PM1	30	39.3	178.2	12.8	2.73	0.37	2.00	15.34	2.06	11.22	551.23[a]	322.34
Pheasant	Raw mince	PM2	30	35.6	178.2	11.8	2.27	0.17	1.53	12.72	0.94	8.61	1537.50[a]	689.79
Pheasant	Raw mince	PM3	30	37.1	185.7	11.0	0.73	0.67	4.80	3.95	3.59	25.85	1391.03[a]	852.60
Pheasant	Tinned meat	PT	30	21.9	87.8	7.6	0.00	0.00	0.07	0.00	0.00	0.76	0.65[b]	0.03
Pheasant	Dry sticks	PS	30	100.0	30.0	15.0	0.00	0.03	1.63	0.00	1.33	54.44	30.79[a]	8.98
Domestic fowl	Raw mince	CM	12	27.9	279.2	10.2	0.00	0.00	0.00	0.00	0.00	0.00	0.09[b]	$
Domestic fowl	Pouches	CP	12	21.3	32.0	6.4	0.00	0.00	0.00	0.00	0.00	0.00	0.46[b]	0.03
Domestic fowl	Dry sticks	CS	11	96.0	48.0	14.7	0.00	0.00	0.73	0.00	0.00	15.15	0.15[b]	$

Table S2. Parameter values from statistical models of the concentration of lead (ppm d.w.) in samples from five petfood products derived principally from the meat of pheasants. The maximum Kolmogorov-Smirnov discrepancy D between the observed and fitted cumulative probability distributions is shown for a model that assumes that the samples of each product came from one log-normal distribution. Superscripts of D values indicate the statistical significance of the maximum discrepancies (+ $P > 0.20$; * $P < 0.05$; ** $P < 0.01$). For each of the three products with a significant discrepancy ($P < 0.05$), results are shown for a mixture model which assumes that the samples for a product were drawn from two groups, each with a different log-normal distribution. The fitted means (M) and standard deviations (S) of the $\log_e$-transformed concentrations for the groups with low and high concentrations are shown, together with the estimated arithmetic mean concentration and the proportion of samples estimated to be in the high group. The expected Poisson probability that a sample would contain at least one shot or radio-dense fragment (large and small), derived from data on the relative masses of samples and package contents and the mean number of objects per package is also shown.

		Low group			High group					
Short name of product	D	M	S	Arithmetic mean (ppm d.w.)	M	S	Arithmetic mean (ppm d.w.)	Proportion of samples in high group	Poisson probability of >0 shot & fragments	Poisson probability of >0 fragments
PM1	0.279*	2.760	0.636	18.57	7.274	1.395	3671.36	0.172	0.306	0.156
PM2	0.350**	2.453	0.411	12.65	8.688	0.696	7562.88	0.200	0.232	0.107
PM3	0.357**	2.515	0.506	14.06	8.96	0.672	9764.78	0.133	0.308	0.277
PT	0.093^{+}	-0.453	0.207	0.65	-	-	-	-	0.006	0.006
PS	0.136^{+}	3.427	0.001	30.79	-	-	-	-	0.565	0.565

Figure S1. Distributions of the concentration of lead (ppm d.w.) in samples from each of five petfood products derived principally from the meat of pheasants. Diagrams show the observed cumulative probability distribution (stepped line) and the expected distribution (smooth curve) from either the fitted two-group log-normal mixture model (products PM1, PM2, PM3) or the single-group log-normal model for the products for which the single-group model gave an adequate fit (PT, PS).

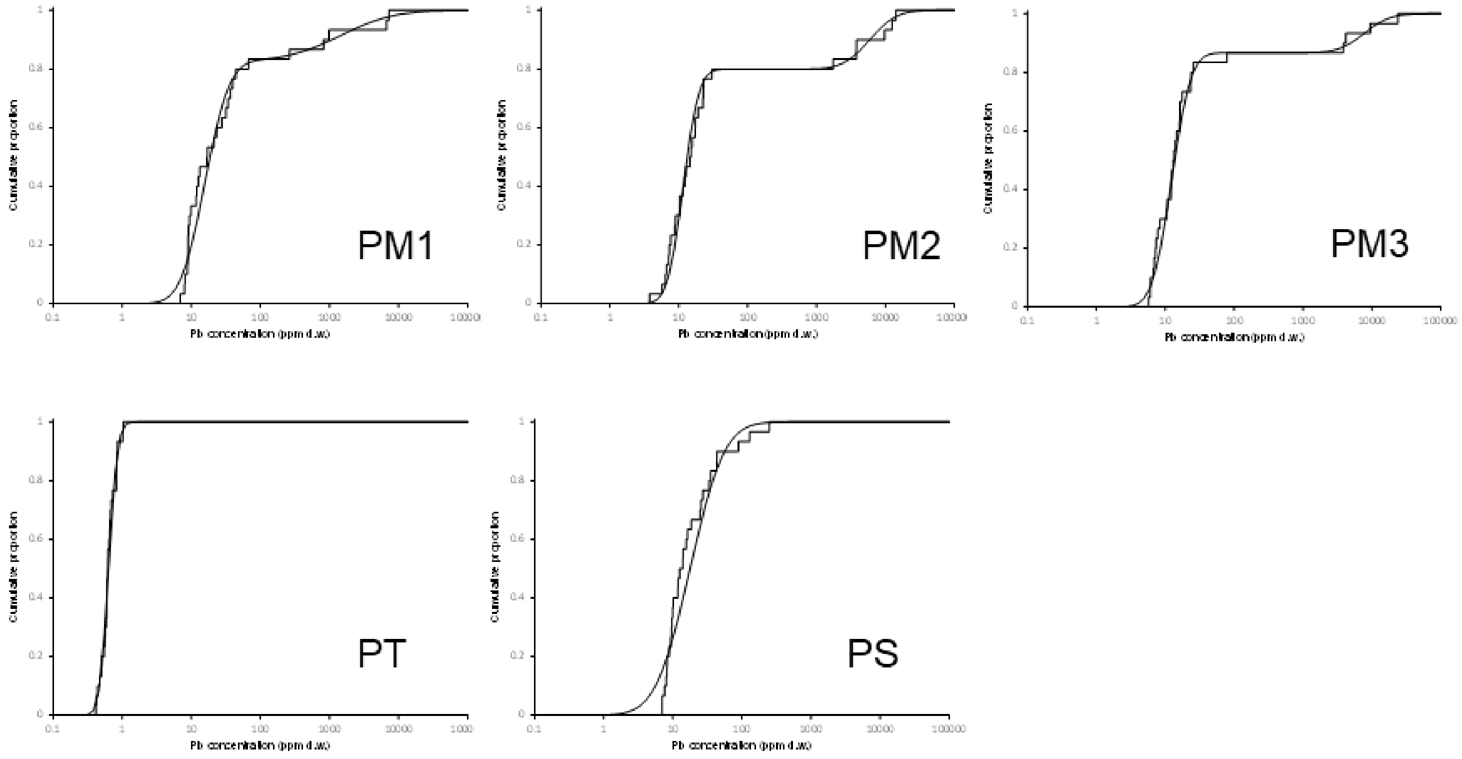

Table S3

Lead concentrations (ppm d.w.) in raw pheasant products marketed for human consumption and as dogfood

Data courtesy of Wild Justice (2021) for pheasant marketed for human consumption and from the current study for raw pheasant dogfood. Samples found by x-ray to contain shot were excluded from the data comparison.

Pheasant meat purchased from three UK food retail outlets Jan-November 2021			Pheasant products (both complete and complementary food) purchased as raw dogfood from three online outlets in January 2022		
0.060	0.482	5.581	3.766	9.947	17.309
0.060	0.538	6.689	5.686	10.322	17.371
0.060	0.569	8.261	5.705	10.331	17.542
0.060	0.660	9.154	6.064	10.484	18.969
0.060	0.745	9.783	6.082	10.780	21.406
0.060	0.773	10.241	6.452	11.552	22.346
0.060	0.777	11.119	6.626	11.806	22.608
0.094	0.797	12.092	6.669	12.068	22.802
0.115	1.138	12.593	6.889	12.091	23.213
0.123	1.756	14.023	6.918	12.133	23.308
0.126	1.941	15.795	7.168	12.250	23.670
0.130	1.959	18.270	7.209	12.500	25.649
0.135	2.014	18.594	7.313	12.634	27.619
0.143	2.100	20.065	7.380	12.780	30.396
0.144	2.278	24.982	7.692	12.991	31.805
0.184	2.476	26.691	7.803	13.285	34.061
0.202	2.765	34.036	7.985	13.419	37.004
0.204	2.782	34.465	8.092	13.989	39.802
0.210	2.858	57.932	8.424	14.491	44.157
0.261	3.243	61.134	8.759	14.519	67.393
0.263	3.299		8.847	15.064	78.889
0.289	3.421		8.972	15.647	262.872
0.319	4.230		8.999	16.061	816.965
0.376	4.345		9.013	16.280	985.790
0.382	4.436		9.048	16.392	1743.877
0.446	4.639		9.103	16.776	3845.077
0.470	4.647		9.473	17.301	9451.238

EU Maximum Residue Levels (MRLs) for lead in animal feed/complementary feed and in complete feed are 10 and 5 ppm w.w. respectively, assuming a moisture content of 12% (EC 2002; for the UK see https://www.legislation.gov.uk/eudr/2002/32); equivalent to 11.36 and 5.68 ppm d.w., respectively.

The EU MRL for lead in the meat (muscle tissue) of domestic stock destined for human consumption is 0.100 ppm w.w. (approximately 0.307 ppm d.w.) (EC 2006). While no level has been formally set for the meat of wild game, similar reporting thresholds are used. 69% of samples marketed for human consumption exceeded the EU MRL of 0.100 ppm w.w. (70% when using the average dry weight conversion as sample moisture varies).

References:

Aebischer, N. J. 2019. Fifty-year trends in UK hunting bags of birds and mammals, and calibrated estimation of national bag size, using GWCT's National Gamebag Census. *European Journal of Wildlife Research* 65(4): 1-13. https://doi.org/10.1007/s10344-019-1299-x

EC 2002. Consolidated Text. Directive 2002/32/EC of the European Parliament and of the Council of 7 May 2002 on undesirable substances in animal feed EUR-Lex - 02002L0032-20191128 - EN - EUR-Lex (europa.eu) Updated 2019 http://data.europa.eu/eli/reg/2019/1869/oj.

EC 2006. COMMISSION REGULATION (EC) No 1881/2006 of 19 December 2006 setting maximum levels for certain contaminants in foodstuffs. Official Journal of the European Union 20.12.2006 L364/5-L364/24 https://www.legislation.gov.uk/eur/2006/1881

Siegel, S. & Castellan, N.J. 1988. Non Parametric Statistics for the Behavioral Sciences, 2nd edn. New York: McGraw-Hill Book Company

Wild Justice. 2021. High lead levels in Waitrose and Harrods game meat. 18th December 2021. https://wildjustice.org.uk/lead-ammunition/high-lead-levels-in-waitrose-and-harrods-game-meat/

Wilkinson, L. 1990. "SYSTAT: the system for statistics. SYSTAT." *Inc., Evanston, IL* 1.
